# A Dual-Modality System for Both Multi-Color Ultrasound-Switchable Fluorescence and Ultrasound Imaging

**DOI:** 10.3390/ijms18020323

**Published:** 2017-02-04

**Authors:** Jayanth Kandukuri, Shuai Yu, Bingbing Cheng, Venugopal Bandi, Francis D’Souza, Kytai T. Nguyen, Yi Hong, Baohong Yuan

**Affiliations:** 1 Ultrasound and Optical Imaging Laboratory, Department of Bioengineering, University of Texas at Arlington, Arlington, TX 76010, USA; jayanth.kandukurijayanth@mavs.uta.edu (J.K.); shuai.yu@mavs.uta.edu (S.Y.); bingbing.cheng@mavs.uta.edu (B.C.); 2 Joint Biomedical Engineering Program, University of Texas at Arlington and University of Texas Southwestern Medical Center at Dallas, Dallas, TX 75235, USA; knguyen@uta.edu (K.T.N.); yihong@uta.edu (Y.H.); 3 Department of Chemistry, University of North Texas, 1155, Union Circle, #305070, Denton, TX 76203, USA; venu235@yahoo.com (V.B.); francis.dsouza@unt.edu (F.D.); 4 Department of Bioengineering, University of Texas at Arlington, Arlington, TX 76010, USA

**Keywords:** ultrasound-switchable fluorescence imaging, ultrasound imaging, dual-modality imaging, multi-color fluorescence imaging, deep-tissue fluorescence imaging

## Abstract

Simultaneous imaging of multiple targets (SIMT) in opaque biological tissues is an important goal for molecular imaging in the future. Multi-color fluorescence imaging in deep tissues is a promising technology to reach this goal. In this work, we developed a dual-modality imaging system by combining our recently developed ultrasound-switchable fluorescence (USF) imaging technology with the conventional ultrasound (US) B-mode imaging. This dual-modality system can simultaneously image tissue acoustic structure information and multi-color fluorophores in centimeter-deep tissue with comparable spatial resolutions. To conduct USF imaging on the same plane (i.e., *x*-*z* plane) as US imaging, we adopted two 90°-crossed ultrasound transducers with an overlapped focal region, while the US transducer (the third one) was positioned at the center of these two USF transducers. Thus, the axial resolution of USF is close to the lateral resolution, which allows a point-by-point USF scanning on the same plane as the US imaging. Both multi-color USF and ultrasound imaging of a tissue phantom were demonstrated.

## 1. Introduction

Simultaneous imaging of multiple targets (SIMT) with high spatial resolution is highly desirable and important in many biological and medical studies in which two or more bio-targets (such as biomarkers, molecules, proteins, cells, blood vessels, nerves/neurons, or signaling pathways) are involved and/or their interactions are interested, such as cancer metastasis, cancer angiogenesis, and neurovascular coupling. For example, when and how cancer cells metastasize into a blood vessel in an in situ tumor is a crucial question for understanding and preventing cancer metastasis. Simultaneous imaging of both cancer cells and blood vessels provides a way to investigate their interactions. Another example is that multiple signaling pathways have been found to possibly regulate angiogenesis in most solid tumors. Therefore, compared with single target therapy, combination therapy, which means simultaneous use of multiple targeting drugs, has great potential to increase the treatment efficiency by simultaneously blocking multiple signaling pathways [1,2]. Accordingly, SIMT can visualize multiple signaling pathways and their interactions [3,4], which can significantly benefit to the investigation of drug resistance mechanisms and the monitoring or evaluation of targeted therapies [1,5,6,7,8,9,10].

Unfortunately, to date, few techniques are available for SIMT [10,11,12]. Fluorescence techniques are highly sensitive and have potential to conduct SIMT based on spectroscopic methods. However, they suffer from poor spatial resolution (a few millimeters) when imaging relatively deep tissue (centimeters) due to strong light scattering of tissue [13,14,15,16,17,18]. Ultrasound, photoacoustic, and fluorescence technologies have been implemented into one system for multi-modality imaging [19,20]. In this type of study, ultrasound waves do not physically interact with fluorescence photons and therefore the spatial resolution of the fluorescence image does not improve. Recently, we developed a new technique, “ultrasound-switchable fluorescence” (USF) based on our recent finding that a focused ultrasound wave can potentially switch fluorescence emission on and off [21]. In USF, the excitation light is delivered into deep tissue via light scattering. When ultrasound is off, the USF agents are off although the excitation light is on. Ideally, no fluorescence is emitted. When ultrasound is on, only the USF agents in the ultrasound focal volume can be switched on to emit fluorescence. The emission photons can propagate out of the tissues via light scattering. Thus, USF can provides fluorescence images of deep tissue with ultrasonic resolution [21,22,23,24,25]. Compared with pure fluorescence techniques, USF overcomes the limitation of the spatial resolution caused by tissue light scattering. Compared with pure ultrasound techniques (US), USF can conduct SIMT based on fluorescence spectroscopy.

This study aims to address the following challenge in USF imaging by developing a dual-modality ultrasound-switchable fluorescence and ultrasound system for both optical and acoustic imaging. Currently, USF can provide in-plane fluorescence images on the *x*-*y* horizontal plane with excellent resolution. This is because its axial resolution along *z* direction, which is the ultrasound wave propagation direction, is ~4–7 times lower than its in-plane lateral resolution (i.e., along *x* or *y* direction). Thus, it degrades the image quality along *z* direction, which is also true for optical microscopy. On the other hand, ultrasound B-mode image shows tissue information on a vertical plane (i.e., *x*-*z* or *y*-*z* plane, so-called tissue cross section plane). In this study, to improve USF axial resolution and be able to co-register USF with ultrasound B-mode imaging, we developed a new dual-modality imaging system via a customized ultrasound transducer that includes two confocal 90°-cross transducers. This new system can simultaneously image tissue cross-section (i.e., *x*-*z* plane) using USF and ultrasound B-mode technologies. We demonstrated that USF simultaneously imaged the distribution of two fluorophores with different excitation and emission spectra for the purpose of SIMT via the optical spectroscopic technology in a tissue phantom. On the other hand, a co-registered B-mode ultrasound image shows the acoustic structure of the tissue phantom. In summary, we improved USF axial resolution, achieved multi-color USF imaging on the cross section plane of the tissue sample for future SIMT, and lastly co-registered USF and B-mode ultrasound imaging for simultaneous dual-modality imaging.

## 2. Dual-Modality Imaging System

### 2.1. Basic Principles of USF and US Imaging

The principle of USF imaging based on fluorophore-encapsulated thermo-sensitive nanoparticles has been described in our recent publications [26,27,28,29]. Briefly, there are two key components in USF imaging: excellent USF contrast agents and a sensitive USF imaging system. Recently, we developed a serial of thermo-sensitive fluorescent nanoparticles [27,28,29]. When encapsulating environment-sensitive fluorophores into a thermo-sensitive nanoparticle, the fluorescence emission shows a switch-like function of the temperature. When the temperature is lower than a threshold (T_th1_), the fluorophores either do not fluoresce or fluoresce very weakly (so-called OFF). When the temperature is higher than another threshold (T_th2_), the fluorophores fluoresce strongly (so-called ON). If the temperature difference between the two thresholds (T_th2_−T_th1_) is narrow (such as a few degrees Celsius), these nanoparticles are called fluorescence switchable contrast agents. When a relatively strong and long ultrasound pulse (200 ms long in this study) is focused into tissue, the temperature in the focal zone can be increased a few degrees Celsius due to the absorption of the acoustic energy. Thus, the contrast agents in the focal zone can be switched on to emit fluorescence, while contrast agents outside the focus remain off. A high-resolution USF image can be generated when scanning the ultrasound focus point-by-point. Note that the delivery of the excitation light and the collection of emission light are based on highly scattered near-infrared photons, USF can image tissues several centimeters deep [26,27,28,29].

In this study, the conventional ultrasound B-mode imaging principle is adopted. Briefly, a relatively short ultrasound pulse (microseconds long in this study) was sent into tissue samples and the echoes reflected from different depths due to the acoustic impedance mismatch were detected. At each location, an averaged A-line was acquired. The envelope profile of this A-line was calculated, which represented tissue’s acoustic information along the depth direction (i.e., *z* direction in this study). By scanning the transducer laterally (i.e., *x* or *y* direction in this study), a serial of A-lines were acquired. Thus, by displaying the envelope profiles of all the A-lines in one figure, a B-mode ultrasound image was generated. Accordingly, a line scanning was conducted for B-mode ultrasound imaging, which is different from USF’s point scanning strategy.

### 2.2. Hardware of the System

**Figure 1:**
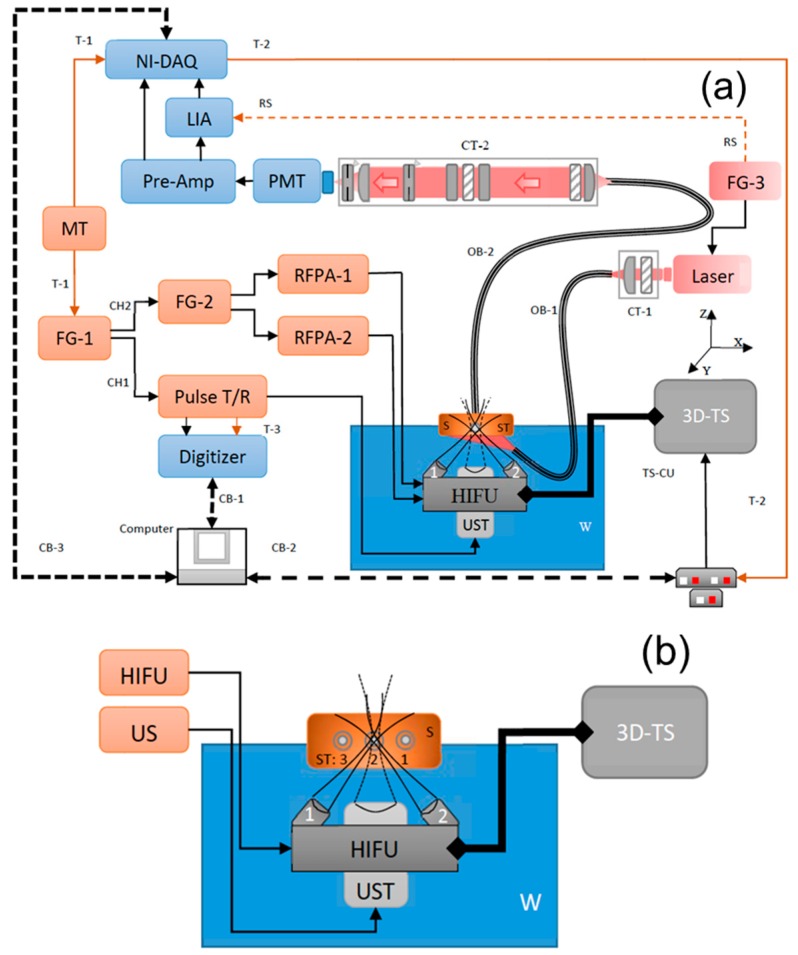

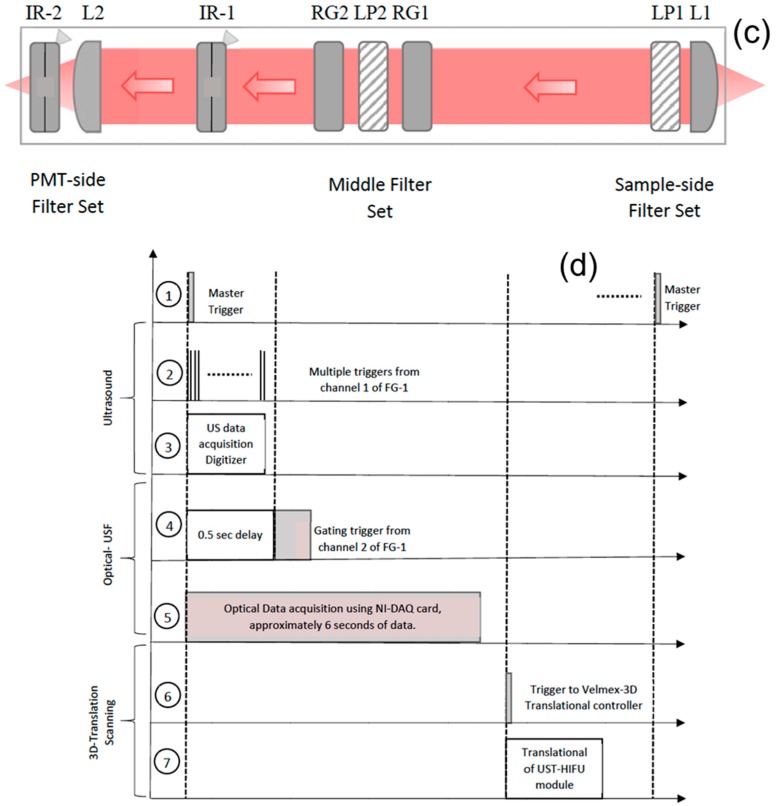
(**a**) Schematic diagram of the dual-modality (Ultrasound B-mode and Ultrasound-Switchable fluorescence) imaging system; (**b**) Schematic diagram depicting the dual-modality setup with dual-confocal focused HIFU with their respective projected ultrasound focuses into sample setup; (**c**) Schematic diagram of optical module of acquisition setup; (**d**) Time sequence event diagram of dual-modality imaging system. MT: Master trigger (T-1) with 0.1-Hz frequency; FG-1: Function generator, Channel-2 for gating (single cycle, pulse signal with 0.5msec delay) and Channel-1 for triggering (1-KHz, 300 cycles, pulse signal) dual-HIFU transducer module (dual-9MHz-HIFU) and Ultrasound transducer (UST) module respectively; FG-2: Function generator for driving each of the dual-HIFU(HIFU-1 and HIFU-2) by means of power amplifier (RFA-1 and RFA-2) respectively using 9MHz sinusoidal signal; Pulse T/R: pulse transmitter and receiver to drive the UST; FG-3: Function generator to modulate, at 1-KHz frequency, the excitation laser source (Laser); W: water tank to immerse the dual-HIFU-UST module and partially immerse the sample (S); ST: Silicone tube of inner diameter (ID): 0.31 mm and outer diameter (OD): 0.64 mm; 3D-TS: three dimensional translational stages; TS-MCU: 3D translational stage motorized control unit; CT-1: collimation tube to focus the excitations laser source into optical bundle (OB-1); CT-2: Optimized collimation tube to guide the collected fluorescence signal from optical bundle (OB-2) from within sample (S); PMT: photo-multiplier tube to detect the optical fluorescence signal; Pre-amp: preamplifier to filter detected optical signal from PMT; LIA: Lock-in amplifier to detect 1-KHz frequency signal from detected optical signal; NI-DAQ: National instrument data acquisition module to record optical signal; Digitizer: National instrument data acquisition module to record ultrasound signal; CB-1: communication bus to transfer ultrasound signal data; CB-2: serial communication bus to control TS-MCU; CB-3: communication bus to transfer optical signal data; T-2: pulse signal with 1-KHz frequency which serves as reference signal to LIA; T-3: single cycle digital pulse signal to trigger the movement of 3D-TS.

The block diagram of the dual-modality imaging system is shown in Figure 1a. It includes three sub-systems: (1) USF for fluorescence imaging; (2) US for B-mode acoustic imaging; and (3) synchronization of USF and US sub-systems.

#### 2.2.1. USF Sub-System

The USF sub-system includes the following modules: (1) an ultrasound heating and its driving module; (2) an excitation light source; (3) a sample module; (4) an optical detection module; and (5) a scanning module.

(1) The ultrasound heating and its driving module.

An ultrasound-heating system (SU-109) was customized and purchased from Sonic Concepts Ltd. (Bothell, WA, USA). It consists of two 90°-crossed and confocally focused ultrasound transducers (Figure 1b). The two transducers were tightly mounted on a base with a 45° angle relative to the horizontal plane of the base (separated 50 mm on the base). Also, their foci were overlapped at their focal planes at a 90° angle (achieved by the manufacturer). The diameter and the focal lengths of each transducer are 23 and 35 mm, respectively. The central frequency is 9 MHz. At the center of the base (between the two transducers) there is a central hollow hole for positioning the B-mode ultrasound imaging transducer that will be introduced in the next section.

To be able to control the driving power of each transducer, the two transducers were driven separately. To do this, a driving signal was generated via two function generators (FG-1 and FG-2) and its power was amplified via two radio-frequency power amplifiers (RFPA1 and RFPA2). First, the channel-2 (CH2) of the FG-1 (AFG 3252, Tektronix, TX, USA) generates a gate signal (i.e., a square pulse). This gate signal is input into FG-2 (Agilent 33500B, Chicago, IL, USA). Thus, two gated 9-MHz sinusoidal waves are respectively generated from the two channels of the FG-2. Their peak-to-peak voltages (*V*_pp_) can be controlled individually. These two signals are amplified by the RFPA-1 and RFPA-2 and then they drive the two transducers, respectively. Note that the gate width of the two gated sinusoidal waves is controlled by FG-1, which determines the exposure time of the two transducers. The peak-to-peak voltages of the two sinusoidal waves are controlled by the two channels of FG-2), which determines the ultrasound exposure power. In this work, the exposure times of the two transducers are kept the same. The driving *V*_pp_ of each transducer is adjusted so that the two transducers roughly have the same exposure power at their foci. Note that the two transducers were found to have different electrical-to-acoustic transfer efficiency, which requires that the transducer with the lower transfer efficiency be driven with higher electrical power (i.e., higher *V*_pp_). To estimate the HIFU-induced temperature rise in its focal volume within the tissue sample, MRI-based thermometry may be needed for an accurate result. However, it is complicated and expensive. Therefore, we adopted a relatively rough but simple method in which the HIFU was focused on the tissue surface and an infrared camera (A325sc, FLIR, Boston, MA, USA) was used to image the temperature. The measured temperature is ~5 degrees Celsius.

(2) The excitation light source.

A continuous wave laser was used as the excitation light source: an 808-nm laser (MGL-II-808-2W, Dragon lasers, Changchun, Jilin, China) for an indocyanine green (ICG)-based USF contrast agent or a 671-nm laser (MLL-FN-671-500mW, Optoengine LLC, Midvale, UT, USA) for another USF contrast agent based on a new fluorophore, aza-BODIPY conjugated with two hydroxyls at the bottom (denoted as ADP(OH)_2_). The excitation light was coupled into a fiber bundle (OB-1, Model # 39366, Edmund optics, Barrington, NJ, USA) and then delivered to the bottom of the tissue sample. The power at the output end of the fiber bundle of OB-1 used in this study are 7.6 and 0.139 mW for 808-nm and 671-nm laser, respectively. For 808-nm laser, the beam is large so that two plano-convex NIR-lenses (AC254-035-B, Thorlabs, Newton, NJ, USA) were used to couple the beam into the fiber bundle. For a 671-nm laser, the beam is small and can be easily coupled into the fiber bundle without using the lenses. In both cases, an optical filter is placed in front of the laser head to attenuate any unknown laser lines from the laser: a band pass filter of 785/62 nm (FF01-785/62-25, Semrock, Rochester, NY, USA) for the 808-nm laser and a band pass filter of 671/11 nm (FF01-673/11-25, Semrock, Rochester, NY, USA) for the 671-nm laser. The other end of the fiber bundle is submerged into water and arranged such that the excitation light illuminates the bottom of the tissue sample but without blocking the ultrasound waves. The intensity of the laser is modulated at 1 kHz via the third function generator (FG-3, 33220A, Agilent, Chicago, IL, USA). The synchronized output (i.e., a 1-kHz square wave) from another channel of the FG-3 is input into the lock-in amplifier (LIA) as the reference.

(3) The sample configuration.

Three silicone tubes (ST, marketed as 1–3 from right to left; 60-011-01, Hellix Medical, Carpinteria, CA, USA) were inserted into a piece of porcine muscle tissue (see Figure 1b). The tissue thickness is around 14 mm and the three tubes with an inner diameter (I.D) of 0.31 mm and an outer diameter (O.D) of 0.64 mm are located in the middle plane. The lateral distance between tube 1 and tube 2 is about 2 mm, and between tube 2 and tube 3 is about 3 mm. The tube 1 and 3 were respectively filled with pure ADP(OH)_2_ and ICG-based USF contrast agent solutions. The tube 2 was filled with a mixed solution of the above two USF contrast agents and the volume ratio between ICG-based solution and ADP(OH)_2_-based solution is 3:2.

(4) The optical detection module.

A second fiber bundle (OB-2, Model # 39366, Edmund optics, Barrington, NJ, USA) was placed on the top of the tissue sample to collect fluorescence emission photons. The other end of this fiber bundle (OB-2) was fixed at the focal point of the NIR plano-convex lens (see L1 in Figure 1c; AC254-030-B, Thorlabs, Newton, NJ, USA) so that the diverging photons from the fiber bundle could be collimated. Another NIR plano-convex lens (L2; AC254-030-B, Thorlabs, Newton, NJ, USA) was placed about 40 cm away from the lens L1 and faced the opposite direction so that the collimated optical beam was focused again on a cooled photomultiplier tube (PMT, H7422-20, Hamamatsu, Bridgewater, NJ, USA). To maximally block the excitation light and pass the emission light, two types of optical filters (interference and absorption filters) were positioned between the two lenses (L1 and L2). First, one interference long pass filter (LP1) was positioned right after the lens of L1 and the second interference long pass filter (LP2) was placed in the middle between the two lenses. Two absorption long pass filters (RG1 and RG2) were put before and after LP2 (Figure 1c). Note that, in each measurement, four filters were used (LP1, LP2, RG1, and RG2), in which LP1 and LP2 are the same type filters and RG1 and RG2 are the same type filters. However, for different contrast agents, the four filters were accordingly changed to match the adopted fluorophore. For ADP(OH)_2_-based USF contrast agents, LP1 and LP2 are 715-nm long pass interference filters (FF01-715/LP-25, Semrock, Rochester, NY, USA), and RG1 and RG2 are 695 nm long-wave pass cut-on filters (FSR-RG695, Newport, Irvine, CA, USA). For ICG-based USF contrast agents, LP1 and LP2 are 830-nm long pass interference filters (BLP01-830R-25, Semrock, Rochester, NY, USA), and RG1 and RG2 are 830 nm long-wave pass cut-on filters (FSR-RG830, Newport, Irvine, CA, USA). An iris (IR-1, SM1D12SZ, Thorlabs, Newton, NJ, USA) was placed between RG2 and L2, and used as a shutter. This iris was adopted mainly for (1) protecting PMT by closing the shutter when adjusting the system; and (2) limiting the background photons by slightly reducing the aperture from its maximum size, as we found that more background photons were distributed around the edge than at the center. A second iris (IR-2; SM1D12SZ, Thorlabs, Newton, NJ, USA) was mounted behind the second lens L2 and in front of the PMT. The aperture was adjusted to ~2 mm (about ~20% of its fully opened aperture) and, functions as a pinhole to block background photons. To further block background photons from the environment, all these components in Figure 1c were mounted into a closed stackable lens tube (SM1 lens tube, Thorlabs, NJ, USA). Finally, the PMT was also mounted on the tube system via a C-mount-to-SM1 convert (SM1A10, Thorlabs, Newton, NJ, USA).

The cooled PMT has an excellent spectral response in the range of 300–890 nm with minimized thermal noise. It was driven by a temperature controlled high-voltage power supply (C8137-02, Hamamatsu, Bridgewater, NJ, USA) and converted the fluorescence signal into an electronic current signal that also has the 1-kHz modulation frequency. A low-noise pre-amplifier (PreAmp; SR570, Stanford Research, Sunnyvale, CA, USA) converted the current signal into a voltage signal and also amplified the signal. The sensitivity of the pre-amplifier is set to 50 nA/V to pass 1-kHz fluorescence signal. Either a 10-kHz low pass filter (with 12 dB/oct rolloff) or a band pass filter (between 3 Hz and 10 kHz, with 6 dB/oct rolloff) could be set from the pre-amplifier to further reduce the noise.

After being processed by the pre-amplifier, the signal was input into a lock-in amplifier (LIA, SR830, Stanford Research, Sunnyvale, CA, USA) for detecting the amplitude of the 1-kHz signal. Note that the synchronized reference signal of the LIA was generated from the FG-3. The output of the LIA provided the dynamic variation of the amplitude of the 1-kHz fluorescence signal and its phase delay relative to the reference. In this study, only the amplitude dynamic variation was needed and acquired. Generally, without ultrasound heating, LIA could also detect a 1-kHz background noise with nearly constant amplitude. This background noise was mainly generated from three sources: (1) laser leakage; (2) auto-fluorescence from the sample; and/or (3) fluorescence from those non-100%-off USF contrast agents. Fortunately, this background 1-kHz noise was independent of ultrasound. When ultrasound heating was applied, the fluorophores in the ultrasound focal region were switched on and then emitted a strong fluorescence signal. Note that the intensity was modulated at 1 kHz because the excitation light was always modulated at 1 kHz. On the other hand, the background noise remained the same as before. Thus, the amplitude of the 1-kHz signal output from the LIA was increased when ultrasound heating was applied. This amplitude change relative to the background was calculated as the USF signal strength. Note that the sensitivity of the LIA was controlled via two parameters, which could be adjusted, time constant and sensitivity. In this study, the time constant was set to 200 ms and sensitivity varied between 20 and 200 mV depending upon the signal strength or the contrast agents. Finally, the LIA signal was acquired by a national instrument data acquisition card (NI-DAQ; PCIE-6363, National Instruments, Dallas, TX, USA) at a sampling frequency of 8 KHz and eventually stored in a computer for offline processing. 

(5) The scanning module.

Three motorized linear translational stages (XN10-0040-E01 series, Motorized XSlide, Velmex, Bloomfield, NY, USA) were orthogonally stacked together to have a 3D scanning capability (3D-TS). The 3D-TS translation stages were controlled using three programmable stepping motor controllers (TS-CU; VXM-3, Velmex, Bloomfield, NY, USA). These TS-CU controllers were connected to the computer via single serial port and were programmed accordingly to perform 1D, 2D, or 3D scanning. A MATLAB GUI was programmed to control these TS-CU controllers, such as step size, acceleration, scan speed, scan plane, number of scan locations within the plane, etc. The three transducers (including the two heating transducers and the one B-mode imaging transducer) were mounted on the base. The base was mounted on the 3D-TS for scanning. All other components remained stationary when scanning was performed.

#### 2.2.2. US Sub-System

In general, ultrasound can provide the acoustic structure information while USF gives the optical information (which may be correlated with molecule events in future applications). If needed, ultrasound can be used to further optimize the localization of the targets in USF imaging. US sub-system was used to acquire A-lines and then form a B-mode ultrasound image (on the *x*-*z* plane). To achieve relatively high resolution, a single element focused ultrasound transducer (UST) with a central frequency of 10 MHz (V315, Olympus, Waltham, MA, USA) was adopted and inserted through the central hollow hole (Figure 1a). The diameter and the focal length of this UST are 19 and 34 mm, respectively. The conventional pulse-echo principle was employed for ultrasound imaging. The UST was driven by a pulser-and-receiver (PR, 5077RP, Olympus NDT, Waltham, MA, USA) that was triggered by the channel 1 of the FG-1. At each scan location, 300 triggers were generated from the FG-1 in 0.3 s to fire 300 narrow high-voltage pulses from the pulser T/R. These high-voltage pulses were converted into 300 acoustic pulses via the UST. These ultrasound pulses propagated in the samples and were reflected by the samples. Eventually, 300 echoes were received by the UST and further amplified by the receiver with 20 dB gain. The signals were acquired by a digitizer with a sampling frequency of 100 MS/s (NI-USB 5133, National Instruments, Dallas, TX, USA). These 300 A-lines were acquired at each scan location and their average was used to form the B-mode image. Note that the number of A-lines at each scan location can be controlled as any number between 1 and 300. In fact, in this study, we found that eight A-lines at each location were enough to acquire a US image with an acceptable signal-to-noise ratio. It is worth pointing out that the USF system requires a point-by-point scanning on the *x*-*z* plane while the US system requires a line-by-line scanning on the same plane. Thus, in this study many abundant A-lines were acquired during the USF point-by-point scanning. Only those A-lines when UST was focused on the tubes were selected to form the B-mode ultrasound image.

#### 2.2.3. Synchronization of USF and US Sub-Systems

Figure 1d shows the time sequences of different events of the entire system. A pulse delay generator with a frequency of 0.1 Hz (DG645, Stanford Research, Sunnyvale, CA, USA) was used as the master trigger (MT, Figure 1a) to trigger both the function generator (FG-1) and the data acquisition card (NI-DAQ). The master trigger (the first row in Figure 1d) initiated the two channels of the function generator FG-1 to send out the triggers (the second row in Figure 1d) for US imaging and the gating pulse (the fourth row in Figure 1d) for USF imaging. Because US imaging is faster than USF imaging, at each scan location multiple A-lines were acquired (the third row in Figure 1d) before firing the heating ultrasound transducer (the fourth row in Figure 1d) for USF imaging. Thus, the gating pulse was delayed 0.5 s relative to the master trigger to make sure enough A-lines were acquired. However, the NI-DAQ was triggered immediately by the master trigger and then acquired 6 s of the data from both the LIA and the pre-amplifier, which included 0.5-s before, 0.2-s during, and 5.3-s after the two heating ultrasound transducers were fired. After both the US and USF data were acquired and stored into a matrix variable using MATLAB, the NI-DAQ was programmed to generate a trigger (see T-2 in Figure 1a) 2 s after finishing acquiring the data, which means a total of 8 s was delayed from the previous master trigger (the sixth row in Figure 1d). Thus, the transducers of USF and US were scanned to the next location (the seventh row in Figure 1d) and then waited for the next master trigger coming for repeating the US and USF data acquisitions. In this study, the scanning is 2D raster scanning on the *x*-*z* plane.

### 2.3. Materials and Synthesis of USF Contrast Agents

The synthesis details of the ICG-based and the ADP(OH)_2_-based USF contrast agents have been introduced in our previous publications [27,29,30]. In this study the switching thresholds for both agents are ~26–27 degrees Celsius and the background temperature is around 23–24 degrees Celsius. Basically, ICG molecules were encapsulated into thermo-sensitive nanoparticles that were made of poly-*N*-isopropylacrylamide (PNIPAM). The synthesis components include *N*-isopropylacrylamide (NIPAM), *N*-tert-butylacrylamide (TBAm), sodium dodecyl sulfate (SDS), *N*,*N*′-methylenebisacrylamide (BIS), *N*,*N*,*N*′,*N*′-tetramethyl ethylene diamine (TEMED), ammonium persulfate (APS), Tetrabutylammonium iodide (TBAI), *N*-(3-Dimethylaminopropyl)-*N*′-ethylcarbodiimide hydrochloride (EDC), and ICG. All these materials were purchased from Sigma-Aldrich Corporate (St. Louis, MO, USA). All chemicals were used as received.

Similarly, ADP(OH)_2_ molecules were encapsulated into thermo-sensitive nanocapsules that were made of Pluronic F98. ADP(OH)_2_ was synthesized based on our earlier published method [31]. Pluronic F98 was obtained from BASF (Florham Park, NJ, USA). Pluronic F98 was dissolved in deionized water with the concentration of 50 mg/mL. The dye/TBAI (molar ratio = 1:6) was dissolved in chloroform and kept in sonication for 30 min. The dye solution was then dropped into the pluronic aqueous solution with stirring and then was dispersed with a sonicator for 4 min. The chloroform was evaporated off to encapsulate the dye into the hydrophobic cores of Pluronic nano-capsules. Free dye was removed using Amicon Ultra centrifugal filters (10,000 molecular weight cut-off, Millipore, Billerica, MA, USA).

### 2.4. Processing of USF and US Data

In this study, to increase the system sensitivity the intensity of the excitation laser is modulated into a sinusoidal wave at 1 kHz [29]. Thus, both the background fluorescence emission and the USF signal are also sinusoidal waves at 1 kHz. Therefore, only the 1 kHz fluorescence signal is acquired, analyzed, and processed, while all other frequency components are rejected. The NI-DAQ acquired the dynamic change of the amplitude of the 1-kHz fluorescence signal before, during and after the heating pulse was applied. The background amplitude was estimated via the data acquired before applying the heating ultrasound pulse, which mainly included laser leakage through the emission filters, possible auto-fluorescence from the sample, and/or fluorescence emission from those non-100%-off USF contrast agents. After applying the heating ultrasound pulse, the amplitude of the 1-kHz fluorescence emission will increase. The difference between the maximum amplitude and the background amplitude was used as the USF signal strength at this location. After scanning, a 2D USF image can be acquired.

For US imaging, A-lines at each scan point were recorded and stored along with the coordinate information such as the location and the distance between the scan point along both the axial and lateral directions. In this study, a total of eight A-lines were averaged at each location to achieve high enough signal-to-noise ratio (SNR), although many more A-lines were available. After all the averaged A-lines were acquired and calculated along the lateral direction (i.e., the *x* direction). A B-mode US image was generated by extracting the envelope of each averaged A-line. The gray scale of the B-mode US image represents the acoustic impedance mismatch between any interfaces in the tissue phantom. It should be noted that US B-mode imaging is a line scan technology so that only a lateral scanning along *x* direction is needed to form a 2D B-mode US image on the *x*-*z* plane. However, USF imaging is a point-by-point scanning technology, so that both lateral and axial scans are needed (i.e., along both *x* and *z* directions). Thus, the system acquired many more A-lines than what was needed to form a B-mode image because of the unnecessary *z*-direction scanning for US imaging. Therefore, some redundant A-lines were discarded.

## 3. Results and Discussion

### 3.1. Single Target

**Figure 2:**
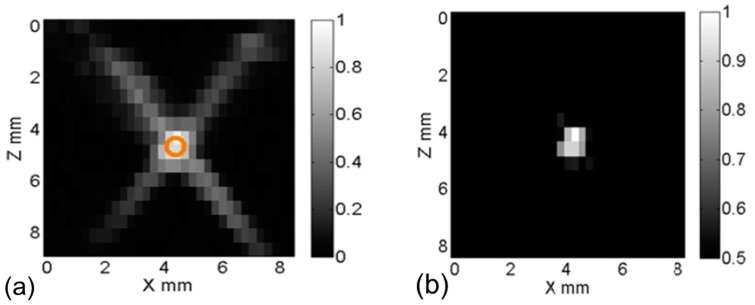
Ultrasound switchable fluorescence images obtained using dual-HIFU for a micro-silicone tube filled with ADP(OH)_2_ based contrast agent; (**a**) with no threshold applied; and (**b**) with 50% and above pass through applied.

We started from the simplest case where a single micro-tube (ID = 0.31 and OD = 0.64 mm) was embedded in a piece of porcine muscle tissue. The tube was filled with the aqueous solution of the ADP(OH)_2_ based USF contrast agent. To acquire a USF image, the overlapped region of the two crossed foci (OR-TCF) was scanned on the *x*-*z* plane. Figure 2a shows the acquired USF image of the cross section of the micro-tube. The circle indicates the location and size of the micro-tube. The gray scale of each pixel on this image indicates the local USF signal strength when the OR-TCF was located at that pixel. Clearly, the USF image not only shows the tube but also shows four long tails. The OR-TCF region shows stronger USF signals than the tails do. This observation agrees to the idea that the OR-TCF region has a higher temperature increase (induced by two heating ultrasound transducers) than the tails (induced by one of the transducers). In fact, these tails are artifacts, which do not represent regions where USF contrast agents are located, and should be shortened as much as possible.

Based on the classical acoustic diffraction theory, the ratio between the axial and lateral focal size is roughly equal to a product between a factor of 7 and the transducer’s *f*-number that can be roughly calculated via the ratio of the focal length to the diameter of the adopted transducer. Currently, the two ultrasound-heating transducers have a relatively large *f*-number (~1.52). Therefore, the tails are relatively long in Figure 2a. To short these tails, using two transducers with an *f*-number smaller than 1 will definitely be helpful. On the other hand, these tails can also be shortened (or even removed) via a few mathematical algorithms. The simplest method is to set a threshold for USF signal strength. Any signal below the threshold will be set as zero.

Figure 2b shows the processed USF image by setting the threshold as 50% of the maximum USF signal. Accordingly, the tail artifacts are successfully shortened. The full width at half maximum (FWHM) of the crossed central region of this USF image is ~1.07 and ~1.5 mm along *x* and *z* directions, respectively. Although these sizes are still larger than the tube outer diameter (0.64 mm), which is mainly caused by the finite acoustic and thermal sizes of the OR-TCF, they are much more uniform along *x* and *z* directions compared with the non-uniform focal size of each individual heating transducer. Specifically, the lateral and axial acoustic OR-TCFs of the SU109 transducer are 0.2 and 0.4 mm respectively, which are specified by the manufacturer. In addition, their corresponding thermal sizes are 0.27 and 0.52 mm, respectively, measured via an infrared camera (the detailed method can be found in our previous publication [32]). However, the lateral and axial acoustic focal sizes are measured using pulse-echo method [33] to be around 0.56 mm and 0.62 mm along lateral and axial, respectively. The reason we selected 50% of the maximum USF signal as the threshold is because two ultrasound beams are used in this study. Ideally, the threshold should be selected close to the inverse of the number of the overlapped ultrasound beams. For example, if four beams are adopted, the threshold may be selected as 25% (i.e., one-quarter). Clearly, when the number of the beams increases, the threshold will be reduced. This is good to avoid artificial errors caused by using the threshold. However, it will become more difficult and complicated in practice. Therefore, the number of the crossed beams should be selected appropriately.

Based on the above results and discussions, the hypothesis that the axial resolution for USF imaging can be improved by using two (or more in future) ultrasound heating transducers with an overlapped focus is demonstrated. Certainly, this threshold-based method may be only good for samples with simple targets. Other methods, such as a morphological recognition method, a de-convolution method, or an ultrasound-guided localization method may be developed for more complicated samples.

### 3.2. Simultaneous Imaging of Multiple Targets Using Dual Modality Imaging

**Figure 3:**
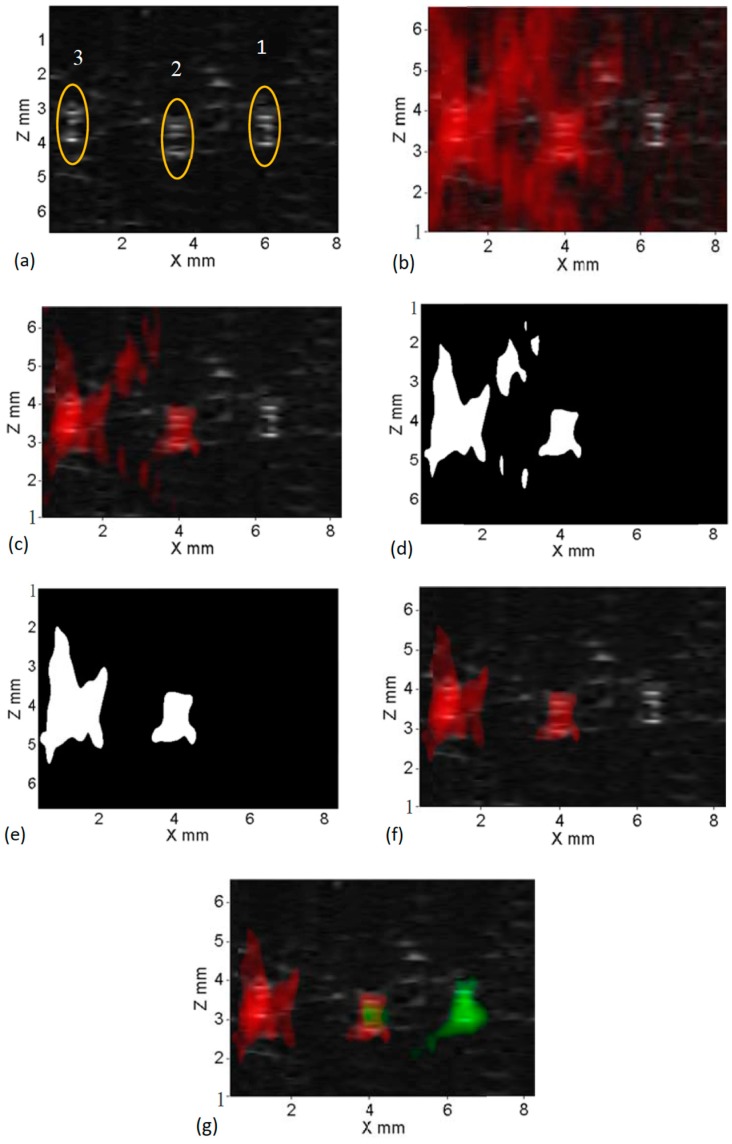
Results obtained using dual-modality imaging system; (**a**) US B-mode image depicting locations of three embedded silicone tubes within a porcine tissue sample; (**b**) USF-ICG image overlaid onto US B-mode image with no threshold applied; (**c**) USF-ICG image overlaid onto US B-mode image with 50% pass through threshold applied; (**d**) binary image obtained by morphological operations; (**e**) final binary image without tail artifacts; (**f**) processed USF-ICG image (using (**e**)) overlaid onto US B-mode image; and (**g**) multi-color (red-ICG and green-ADP(OH)_2_ contrast agent) multi-modality processed image.

In this section, three tubes (see the configuration in Figure 1b) were imaged using this dual-modality system. Tube 1 and 3 were filled with ADP(OH)_2_ and ICG-based USF contrast agent, respectively. Tube 2 was filled with the two mixed solutions with a volume ratio of 3:2. Figure 3a show their US B-mode image. The three tubes can be clearly localized as indicated by the three circles as depicted by yellow circles in Figure 3a (no matter which solutions were filled). This is because US is sensitive to the difference in the acoustic impedance between the tube material and the surrounding porcine muscle tissue. However, US is insensitive to the fluorophore types so that it cannot differentiate which fluorophore is filled in each tube.

To differentiate the fluorophores, ICG-based USF contrast agents were first imaged using the USF system. An 808-nm laser was used as the excitation light source. The emission filters included two 830-nm long pass interference filters and two RG830 absorption filters. Figure 3b shows the USF image (overlaid on the US image) without applying any thresholds. Figure 3c show the similar images with a threshold of 50% of the maximum USF signal. It can be seen that the majority of the tail artifacts can be removed (although not all). After being processed with this threshold method, we realized that some remaining artifacts that were separated from the main central region (i.e., the tube region) could be further removed. The method was to convert the USF image in Figure 3c into a binary image (see Figure 3d). Any disconnected areas from the main central region were set as zero. Thus, a new binary image was generated and shown in Figure 3e. Then, multiplying this new binary image with the USF image shown in Figure 3c, most artifacts can be removed. The result is shown in Figure 3f. Besides finding the locations of the tube 2 and 3, more important result is that only tubes 2 and 3 that were filled with ICG-based contrast agents are observed from the USF image. The tube 1 that was filled only with ADP(OH)_2_-based contrast agents was not shown. This is because the 808-nm laser does not excite the ADP(OH)_2_.

To image ADP(OH)_2_-based USF contrast agents in tube 1 and 2, another USF scanning was conducted using a 671-nm excitation laser and a set of emission filters (two 715-nm long pass interference filters and two RG695 absorption filters). After processing the data using the similar method described above and the method described in our previous publication, the USF signals from the ADP(OH)_2_-based USF contrast agents are overlaid on Figure 3f and the final image is shown in Figure 3g. By comparing Figure 3a and Figure 3g, the three tubes are clearly located by the US B-mode image and roughly located by the USF image. The fluorophores of ICG, ADP(OH)_2_ and their mixture are clearly resolved via different colors (red and green), which cannot be achieved by the B-mode image. Accordingly, this dual-modality imaging system combines our color-sensitive USF imaging with a conventional B-mode US imaging to provide both the acoustical structural information (such as the location, shape, size, depth, etc., of the target) and the USF functional, biochemical, or molecular information.

## 4. Conclusions

In conclusion, we developed a dual-modality imaging system by combining our recently developed USF imaging with the conventional ultrasound B-mode imaging. This dual-modality system has the several unique features. (1) By using two 90°-crossed ultrasound transducers with an overlapped focal region, the axial resolution (along the ultrasound wave propagation direction) of USF imaging has been significantly improved (close to its lateral resolution), which makes it possible to scan tissue on the *x*-*z* plane co-registered with a B-mode ultrasound image. In addition, it is helpful for developing 3D USF imaging in future; (2) By combining the two imaging modalities, the system can image multi-color fluorophores in tissues via the USF technology and also image tissue acoustic structures via the B-mode ultrasound. Simultaneous imaging of multiple targets (SIMT) is an important goal for molecular imaging in the future. Therefore, this dual-modality imaging technology provides great potentials for achieving this goal.
